# Temporal pain processing in the primary somatosensory cortex and anterior cingulate cortex

**DOI:** 10.1186/s13041-022-00991-y

**Published:** 2023-01-05

**Authors:** Guanghao Sun, Michael McCartin, Weizhuo Liu, Qiaosheng Zhang, George Kenefati, Zhe Sage Chen, Jing Wang

**Affiliations:** 1 Department of Anesthesiology, Perioperative Care and Pain Medicine, New York University Grossman School of Medicine, New York, NY 10016 USA; 2 Department of Psychiatry, New York University Grossman School of Medicine, New York, NY 10016 USA; 3 Interdisciplinary Pain Research Program, New York University Langone Health, New York, NY 10016 USA; 4 Department of Neuroscience & Physiology, New York University Grossman School of Medicine, New York, NY 10016 USA; 5 Neuroscience Institute, New York University Grossman School of Medicine, New York, NY 10016 USA

## Abstract

Pain is known to have sensory and affective components. The sensory pain component is encoded by neurons in the primary somatosensory cortex (S1), whereas the emotional or affective pain experience is in large part processed by neural activities in the anterior cingulate cortex (ACC). The timing of how a mechanical or thermal noxious stimulus triggers activation of peripheral pain fibers is well-known. However, the temporal processing of nociceptive inputs in the cortex remains little studied. Here, we took two approaches to examine how nociceptive inputs are processed by the S1 and ACC. We simultaneously recorded local field potentials in both regions, during the application of a brain-computer interface (BCI). First, we compared event related potentials in the S1 and ACC. Next, we used an algorithmic pain decoder enabled by machine-learning to detect the onset of pain which was used during the implementation of the BCI to automatically treat pain. We found that whereas mechanical pain triggered neural activity changes first in the S1, the S1 and ACC processed thermal pain with a reasonably similar time course. These results indicate that the temporal processing of nociceptive information in different regions of the cortex is likely important for the overall pain experience.

## Introduction

Pain is a highly salient signal, and acute pain in many ways protects us from injury and harm. Unlike other sensory modalities, however, there is no single target in the brain for pain representation [1–5]. In contrast, neuroimaging studies in human subjects have demonstrated a highly complex, distributed network of pain-processing regions [6–10]. Despite the overall complexity of this network, several cortical regions within this network appear to play key roles. The primary somatosensory cortex (S1), for example, is known to encode the sensory-discriminative aspect of pain, including the location and quality of pain, whereas the anterior cingulate cortex (ACC) has been shown to play an important role in the affective or aversive response to pain [1–3, 11–14].

MRI and PET studies in humans have provided reasonably detailed anatomic view of various brain structures and enabled functional connectivity studies to understand how sensory information flows from one area of the brain to the next. However, these study modalities do not permit investigation of highly temporally-regulated events. In contrast, invasive neurophysiological recordings can be used to detect events that occur on the order of milliseconds. Prior work using invasive recordings in animal models has shown how neurons in various brain regions process nociceptive inputs [13, 15–23]. However, in most of these studies, a single region of the brain was targeted. As a result, we do not yet know in detail the temporal order of pain processing among different cortical regions. Such temporal order, however, is critical for understanding how the nociceptive information is either directly or indirectly transferred from one cortical region to the next. This information flow can dictate the integration of the sensory and affective pain experiences, which in turn gives rise to the overall pain experience.

Local field potentials (LFPs) combine synaptic and network activities within a recorded brain region. They represent aggregate subthreshold activity which indicate input information in a local brain region [24]. Since LFPs measure the collective behavior of ensembles of neurons, frequency-specific LFPs are thought to process distinct network information. For example, theta oscillation (4–8 Hz) in the hippocampus is known to be involved in the formation of memory in rodents [25, 26]. Among different cortical rhythms, gamma oscillation (30–100 Hz) is particularly known to be important for sensory processing in the cortex [24], and gamma band activities in the cortex have been shown to play a role in pain perception [27–34]. We have recently developed a closed-loop brain-computer interface (BCI) that links pain-related LFP signals from the S1 and ACC with neurostimulation of the prefrontal cortex to deliver temporally precise detection and treatment of pain [35, 36]. The ability of this BCI in treating pain indicates the success of pain detection using a machine learning algorithm that is based on analysis of the higher frequency LFP signals, including gamma band oscillations [35, 36].

In this study, we have used this BCI system to record neural activity from the S1 and ACC in response to mechanical and thermal noxious inputs in freely moving rats. We have analyzed event-related potentials (ERPs) which are native neurophysiological responses to stimulus inputs, and we have also analyzed pain decoder function in the S1 and ACC. We found that the pain decoder detected pain first in the S1, followed by detection in the ACC, in response to a mechanical pain stimulus. In contrast, both regions detected slowly ramping thermal pain at approximately the same time. These results suggest that nociceptive information arrives at relevant cortical areas at different times, and that the modality of peripheral stimulus may also influence cortical nociceptive processing. This temporally sensitive processing of nociceptive information in different regions of the cortex is likely important for the overall pain experience.

## Materials and methods

### Experimental protocol and data acquisition

All experimental studies were conducted in accordance with the New York University School of Medicine (NYUSOM) Institutional Animal Care and Use Committee (IACUC) regulations to ensure minimal animal use and discomfort, license reference number: IA16-01388. Male Sprague–Dawley rats were purchased from Taconic Farms and kept in a rearing room facility in the NYU Langone Science Building, controlled for humidity, temperature, and a 12-h (6:30 a.m. to 6:30 p.m.) light–dark cycle. Food and water were available ad libitum. Animals arrived at the facility weighing 250 to 300 g and had an average of 10 days to acclimate to the new environment before the experiment began.

### Silicon probe implantation surgery

Two 32-channel silicon probes (Buzsaki32-H32, NeuroNexus Technologies, or ASSY-116 E-1, Cambridge NeuroTech) were used for neural recordings. One probe was used for the ACC recordings and the other for S1 recordings. During the surgery, rats were anesthetized with isoflurane (1.5 to 2%). The silicon probes were implanted in the S1 (AP − 1.5 mm, ML ± 3.0 mm, DV − 1.1 mm with angle 0°) and the ACC (AP + 2.7 mm, ML ± 1.6 mm, DV − 2.0 mm with angle 20°). After the electrodes were implanted, we used silicone artificial dura gel (Cambridge NeuroTech) to protect the dura. The drives of the silicon probe were fixed to the skull screws with dental cement. After surgery, the rats were given several days of recovery time before neural recordings.

### Event-related potential (ERP) analysis

Event-related potentials (ERPs) are also called "evoked potentials" when they occur shortly after a stimulus. The cortical ERP reflects the coordinated behavior of a large number of neurons related to external or internal events. Traditional ERP analyses are based on trial averaging, but the ERP statistics we report here are based on a single-trial analysis. Since ERP is associated with low-frequency activity, we further used band-pass filtering (4–100 Hz) to the multi-channel LFP trace to remove high-frequency noise. In order to better compare with the subsequent Z-score peak latency, we chose the channels used to calculate the Z-score and calculated the average trace of selected channel LFP data for extracting ERP latency. To calculate the ERP latency, in mechanical stimulus (PP) tasks, we identified 1 s data from each trial, with 0.5 s before PP as baseline and 0.5 s after PP as cortical nociceptive response. In thermal stimulus (Hargreaves) tasks, we identified 0.5 s before thermal stimulus onset as baseline and between the thermal stimulus onset and paw withdrawal as cortical nociceptive response. Next, the mean and standard deviation of baseline was calculated. The ERP latency was defined as the time interval between the stimulus onset and peak response. Furthermore, if there was more than 1 peak identified and these peaks were above mean + 3*std, we replotted the trace and manually selected the first peak for ERP latency.

### Unsupervised machine learning analysis for detecting the onset of pain signals

For the LFP features, we used band-pass filtering to extract the band-limited signal and detect the low gamma (30–50 Hz), high gamma (50–100 Hz) and ultra-high frequency ranges (300–500 Hz). The frequency range > 300 Hz is also known as spiking-band power or multi-unit activity. The features are averaged by bin size to generate a 3D time series for a single selected LFP channel. Criteria for channel selection are based on artifacts, signal-to-noise ratio (SNR), or spike activity.

We use an unsupervised machine learning method to detect the pain onset. We developed this decoding method based on a state-space model (SSM), a type of linear dynamical system. The SSM has two components: the state equation and the measurement equation. In the state equation, we assume that the ACC or S1 spectral-temporal features (amplitudes at 30–50 Hz, 50–100 Hz and 300–500 Hz) at the *k*-th time index (bin size: 100 ms), are represented by the vector ${{\varvec{y}}}_{k}$, driven by a univariate latent Markov process ${z}_{k}$:

$${z}_{k}=a{z}_{k-1}+{\epsilon }_{k}$$

where ${\epsilon }_{k}$ specifies a temporal Gaussian prior (with zero mean and variance σ^2^) on the latent process, and 0 <*|a|*< 1 denotes the first-order autoregressive coefficient.

In the measurement equation, we assumed that the measurement ${{\varvec{y}}}_{k}$ was drawn from a linear Gaussian system

$${{\varvec{y}}}_{k}={\varvec{c}}{z}_{k}+{\varvec{d}}+{\varvec{v}}$$

where ${\varvec{d}}$ is a constant; ${\varvec{c}}$ is the modulation coefficient; and ${\varvec{v}}$ is an uncorrelated Gaussian noise with zero mean and a covariance matrix **Σ**. The latent variable ${z}_{k}$ was seen as a common input driving the pain response in ${{\varvec{y}}}_{k}$.

We set all unknown model parameters to Θ and develop an iterative expectation maximization (EM) algorithm to estimate the latent states $\{{z}_{k}\}$ (E-step) and the unknown parameters Θ = {*a, c, d*, σ^2^, **Σ**} (M-step). During the computation we use a Kalman filter to estimate the predicted latent state. The Kalman filter equation is as follows:

$${\widehat{z}}_{k|k-1}={a\widehat{z}}_{k-1|k-1}$$

$${Q}_{k|k-1}={{a}^{2}Q}_{k-1|k-1}+{\sigma }^{2}$$

$${\widehat{{\varvec{y}}}}_{k|k-1}={{\varvec{c}}\widehat{z}}_{k|k-1}+{\varvec{d}}$$

$${{\varvec{G}}}_{k}={Q}_{k|k-1}{{\varvec{c}}}^{T}{({Q}_{k|k-1}{\varvec{c}}{{\varvec{c}}}^{T}+{\varvec{\Sigma}})}^{-1}$$

$${\widehat{z}}_{k|k}={\widehat{z}}_{k|k-1}+{{\varvec{G}}}_{k}({{\varvec{y}}}_{k}-{\widehat{{\varvec{y}}}}_{k|k-1})$$

$${Q}_{k|k}={Q}_{k|k-1}(1-{{\varvec{G}}}_{k}{\varvec{c}})$$

where the subscripts *k*|*k*-1 and *k*|*k* are the estimated values of the prediction and filtering operations. ${\widehat{z}}_{k|k}$ and ${Q}_{k|k}$ denote the posterior mean and variance of the latent state, respectively; ***G***_*k*_ denotes the Kalman gain. In summary, recursive updates between the prediction and filter equations yield sequential Bayesian estimates of latent state ${\widehat{z}}_{k|k}$. We further compute the Z-score of the state: $Z\mathrm{ score}=\frac{z-\mathrm{mean}({z}_{\mathrm{baseline}})}{\mathrm{SD}({z}_{\mathrm{baseline}})}$. The significance criterion for the change in Z-score was determined by the critical threshold. We used a 95% confidence interval when the lower bound Z-score is greater than 3.38 (Z-score − CI > 3.38) or the upper bound Z-score is less than − 3.38 (Z-score + CI <  − 3.38), where the confidence interval (CI) for each time point was from the state posterior variance ${Q}_{k|k}$.

### State Z-score peak latency analysis

Similar to ERP latency, in mechanical stimulus (PP) tasks, we identified 0.5 s after PP as cortical nociceptive response. In thermal stimulus (Hargreaves) tasks, we identified between the thermal stimulus onset and paw withdrawal as cortical nociceptive response. The Z-score peak latency was defined as the interval between the stimulus onset and absolute Z-score peak value time in response.

### Statistical analysis

Neural and behavioral data were analyzed offline by custom MATLAB (version 2018, MathWorks) scripts and GraphPad Prism version 8 software (GraphPad). All results are reported as mean ± SEM. Comparisons between the means of the two groups were evaluated by the two-tailed paired t-test. Differences were considered statistically significant when p < 0.05. Exact p-values and sample sizes are shown in figure legend.

## Results

### Using a multi-region BCI to detect pain signal

**Fig. 1:**
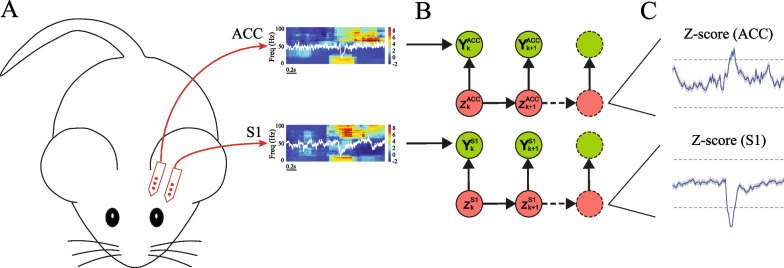
Design of a multi-region LFP-based neural interface for pain. **A** We recorded local field potentials (LFPs) form the rostral area of the anterior cingulate cortex (ACC) and primary somatosensory cortex (S1) using silicon probe arrays in rats. **B** Raw LFP signals were processed to compute three band-limited LFP power features ${\{\mathbf{Y}}_{k}^{\mathrm{ACC}}\}$ and ${\{\mathbf{Y}}_{k}^{\mathrm{S}1}\}$ form ACC and S1 channels (low gamma (30–50 Hz), high gamma (50–100 Hz), and ultra-high frequency (300–500 Hz)), and sent to an automated decoder based on a state space model (SSM), which independently inferred the latent variables ${\{z}_{k}^{\mathrm{ACC}}\}$ and ${\{z}_{k}^{\mathrm{S}1}\}$. **C** In the presence of noxious stimulus, the SSM determined the relative change in Z-score from baseline and used this change as a proxy for acute pain signals

Our prior studies have shown that features of LFP in the low-gamma (30–50 Hz), high-gamma (50–100 Hz), and ultra-high-frequency (300–500 Hz) bands are highly specific to pain processing [16, 34, 37]. Thus, we have used amplitudes from these frequencies to design a pain decoder that is based on a modified state space model (SSM). We used frequency data from LFPs recorded in the hind limb S1 region and the rostral ACC. We then designed a BCI to link this decoder with stimulation of the prelimbic cortex, a brain region that is known to produce descending pain regulation [38–43] (Fig. 1A). We applied this BCI to detect and treat pain during noxious stimulation of the contralateral hind paw of rats with either a mechanical (pinprick, PP) or thermal stimulus (infrared emitter from a Hargreaves’ table). Frequency-dependent LFP power features from the S1 and ACC were computed and sent into an SSM-based online decoder (Fig. 1B). In the presence of a noxious stimulus, the SSM determined the relative change in observed neural activity (Z-score) from baseline and used Z-score values as a proxy for the acute pain signal (Fig. 1C). In prior studies, we have tested the accuracy of pain detection and found it to be as high as 90% [35, 36]. The accuracy of this automated pain detector was further tested in combination with therapeutic optogenetic or electrical stimulation of the prelimbic cortex in the design of a pain BCI, and it was found that this BCI, driven by the pain decoder, inhibited pain behavior with high efficacy [35, 36].

### Stimulus-evoked ERPs and algorithmically driven pain detection identified changes in the S1 prior to ACC in response to mechanical noxious inputs

**Fig. 2:**
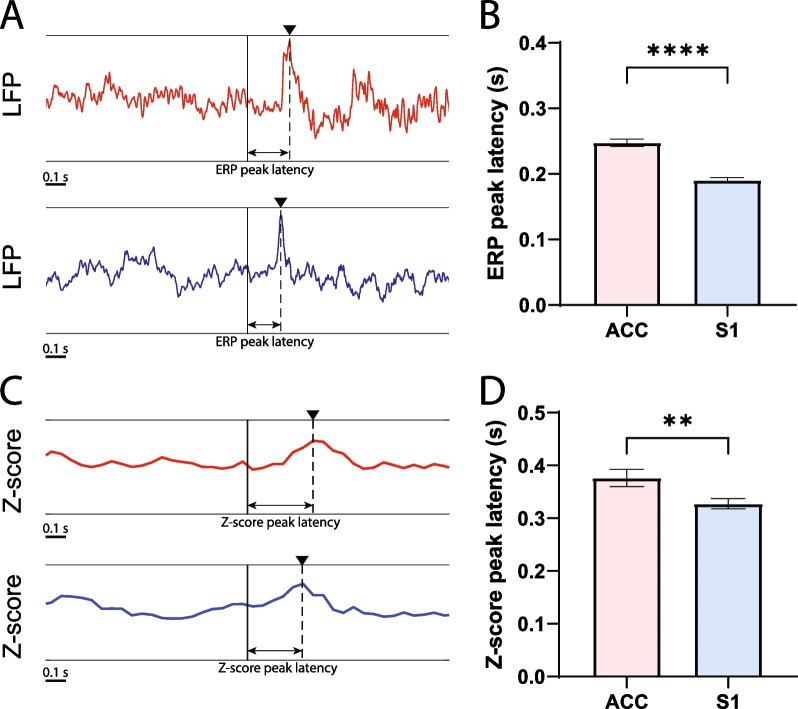
Pain-evoked event-related potentials (ERPs) and Z-score analysis of the pain decoder showed temporal sequence in cortical response to a mechanical stimulus. **A** Illustration of raw LFP trace of ACC and S1. ERPs are marked by black triangles. Onset of a noxious mechanical stimulus (pin prick, PP) is marked by the black vertical line. The red curve indicates LFP signals in the ACC and the blue curve indicates LFP signals in the S1. The arrow marks the time of the ERP peak latency. **B** Comparison of the ERP peak latency between the ACC and S1 (n = 96 trials from 5 rats). On average, the ERP peak latency in the ACC was longer than that of the S1 (n = 96; ****p < 0.0001, paired* t* test). **C** Illustration of Z-score curve of the SSM-based pain decoder in the ACC and S1 (see Methods for details). Z-score peak is marked by the black triangle. The onset of PP is marked by the black vertical line. The red curve indicates the Z-score curve in the ACC and the blue curve indicates Z-score curve in the S1. The arrow marks the time of the Z-score peak latency. **D** Comparison of Z-score peak latency between the ACC and S1 (n = 96 trials from 5 rats). The latency in the ACC was longer than the latency in the S1 (n = 96; **p = 0.0014, paired* t* test)

We first analyzed ERPs and pain decoder data in rats that underwent mechanical noxious stimulations. We identified pain-evoked ERPs from the raw LFP traces in the ACC and S1 (Fig. 2A). Here we set the time from the onset of the PP stimulus to the peak of the ERP as ERP peak latency. We then compared ERP peak latency in the S1 with ERP latency in the ACC during each trial. We found that the ERP peak latency of the ACC was consistently longer than the latency of S1 (Fig. 2B). Next, we analyzed the timing of pain detection using the automated decoder of ACC and S1. Similar to the ERP peak latency, we set the time from the onset of the PP stimulus to the peak of the Z-score curve as Z-score peak latency (Fig. 2C). Again, we found that the pain decoder, which is based on amplitudes from a combination of low-gamma (30–50 Hz), high-gamma (50–100 Hz) and ultra-high-frequency oscillations, demonstrated a similar trend, where the Z-score peak latency in the ACC was longer than the latency in the S1 (Fig. 2D). Together, these results from ERP and Z-scored pain decoder data strongly suggest that mechanical nociceptive information arrived at the S1 before the ACC.

### Z-score detection show similar timing in the response in S1 and ACC to slowly ramping thermal nociceptive inputs

**Fig. 3:**
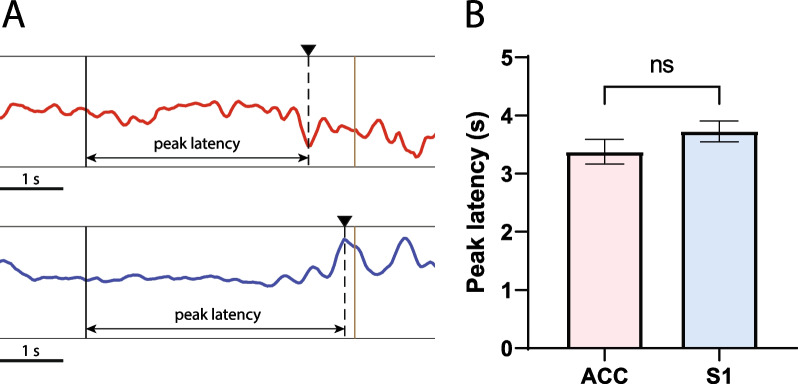
Pain-evoked ERPs and pain decoder did not show clear temporal sequence in cortical processing of a thermal pain stimulus. **A** Illustration of Z-score curve of the ACC and S1. The onset of the thermal stimulus is marked by the black vertical line, whereas the paw withdrawal time is marked by the brown vertical line. We set the maximum absolute value of the Z-score curve between the thermal stimulus onset and paw withdrawal as the Z-score peak value, which is marked by the black triangle. The red curve indicates the Z-score curve in the ACC, and the blue curve indicates the Z-score curve in the S1. The arrow marks the time of the Z-score peak. **B** There is no significant difference in the Z-score peak latency between the ACC and S1 (n = 48 trials from 5 rats; p = 0.1551, paired* t* test)

We next analyzed the timing of cortical processing of thermal nociceptive inputs. We used a Hargreaves’ table to produce thermal pain. In contrast to mechanical pain, which produced nocifensive withdrawals within several hundred milliseconds, thermal pain induced by the infrared emitter produced pain with a more gradual onset (nocifensive withdrawal occurred at approximately 4 s), likely due to the relatively slow ramping up of temperature at the paw tissue. Likewise, due to the slow ramping up of temperature, there were no obvious ERPs as in the case of acute mechanical pain. We then analyzed the decoder function by setting the time from thermal stimulus onset to Z-score peak, which is the maximum absolute value of the Z-score value between stimulus onset and paw withdrawal, as Z-score peak latency (Fig. 3A). Interestingly, unlike the case with mechanical pain, here we found no significant difference in the pain detection latency between the ACC and S1 in the presence of slow-onset thermal pain (Fig. 3B).

## Discussion

In this study, we have measured simultaneous LFP signals from the S1 and ACC of freely moving rats. We found that ERPs triggered by mechanical noxious inputs occurred in the S1 prior to their occurrence in the ACC. We constructed a pain decoder based on changes in amplitudes of gamma and ultra-high frequency signals from LFPs, and found that similarly this pain decoder detected pain first in the S1 and then in the ACC. In contrast, our automated pain decoder did not clearly demonstrate temporal sequence of thermal pain signals in the cortex. These results indicate that mechanical pain signals are likely processed by the sensory cortex prior to processing by prefrontal systems, and that the modality of peripheral pain stimulus may influence the timing of cortical processing.

Whereas spikes are used to provide specific information regarding individual neuronal function, they are difficult to record stably over long periods of time. In contrast, LFPs measure ensemble neuronal activity, and they have been shown to be stable over long periods of time, on the scale of weeks and months. Frequency-specific LFPs are thought to process distinct network information. In particular, high-gamma (60–100 Hz) has been shown to correlate with spike synchrony and could be utilized as a substitute for assessing output of neuronal activity. Moreover, neural oscillations in broadband power spectrum have been shown to be involved in a number of pain states [33, 44–46]. Thus, in our study, we have chosen to use higher frequency range data in the design of a pain decoder and use this decoder to analyze the timing of cortical responses to nociceptive inputs. We have had success in decoding and treating pain when using this decoder in a therapeutic BCI design from prior studies [35, 36].

The cortex does not have a single region that specifically processes pain. Instead, different cortical areas process different aspects of pain. Two prominent regions for pain processing are the ACC and S1. Whereas the ACC is known for processing the affective component of pain [1–4, 13, 15, 16, 18, 19, 47–49] (neural activity in this region has been used to decode the intensity and timing of pain [13, 15–17]), S1 provides critical sensory information for pain in a somatotopic manner. The S1 is known to receive nociceptive inputs from the lateral thalamus, whereas the ACC receives inputs from the medial thalamus [20, 50, 51]. Our results for mechanical pain indicate that the S1 at least in some instances may receive nociceptive signals prior to the ACC. These results in turn suggest the possibility that nociceptive information from the S1 can then be further transmitted to the ACC. Indeed, two previous studies have shown the possibility of this information transfer [17, 52]. In one of these studies, ACC neurons that receive nociceptive inputs from the S1 are shown to play a larger role in aversion processing than other ACC neurons, and that S1-ACC projection is involved in the maladaptive plasticity in the ACC that is responsible for enhanced aversion in the chronic pain state [17].

There are a number of potential interpretations for why the S1 did not demonstrate an earlier timing for thermal nociceptive processing than the ACC in our study. First, it could be due to the nature of peripheral and spinal neural transmission. Sharp, well-localized pain such as that occurring from mechanical nociceptive stimulus is known to be conducted by A-delta fibers which are myelinated and fast-conducting. In contrast, diffuse pain produced by a thermal stimulus from our infrared emitter is likely mediated by c fibers which are unmyelinated and slower-conducting. It is quite possible that A-delta and c fiber have not only different rates of conduction, but also different projections centrally to the thalamus and subsequently to the cortex [53, 54]. Furthermore, there is a possibility that pin pricks can also produce a touch sensation in addition to pain, and thus may also trigger A-beta fiber conduction which conducts faster than A-delta and c fibers. This is less likely, however, as prior studies have shown that non-noxious von Frey filaments did not trigger similar ERP or automated pain detection as pin pricks [35, 36]. At the molecular level, mechanical and thermal stimuli are known to activate different classes of nociceptive receptors at nerve endings, which may further contribute to different ascending nociceptive input speeds [55–57]. In addition to peripheral and spinal mechanisms, there are also more central potential explanations for why the S1 did not demonstrate an earlier timing for nociceptive processing than the ACC. It is possible that due to the slow ramping speed of thermal pain, there are subthreshold activities in both the S1 and ACC. These subthreshold activities may have occurred during the ramping up period leading to the threshold event that immediately preceded pain detection. These subthreshold activities may have primed the cortical system to more quickly respond to cross-threshold nociceptive inputs from the thalamic nuclei, erasing the potential timing difference in nociceptive processing between the S1 and the ACC that is seen with the quicker onset mechanical nociceptive stimuli. Such cortical information priming is known to occur in various types of sensory processing [58–60].

LFPs share many similarities with electroencephalogram (EEG) signals, and thus studies of LFPs in animals have a potential for translation to human EEG studies [24]. In particular, there are findings of increased gamma oscillations in response to noxious stimuli [29, 30]. Recent technical development further enables a single EEG electrode to record temporal-spectral pattern over single-trial stimulations and provide information about neuronal responses to noxious stimuli [28]. Thus, future studies to adapt similar analysis to EEG signals may provide understanding of cortical nociceptive processing in humans.

In summary, we have shown that LFP signals can be used to detect the onset of pain processing by cortical regions. These inquiries allow us to understand not only the timing of nociceptive information processing but the possibility of nociceptive information flow in the cerebral cortex. Extensions of this kind of analysis can be applied to other cortical as well as subcortical areas to further study how pain is processed in the brain.

## Data Availability

Data associated with this study are present in the paper or available upon reasonable request.

## Abbreviations

ACC: Anterior cingulate cortex
BCI: Brain-computer interface
CI: Confidence interval
EEG: Electroencephalogram
EM: Expectation maximization
ERP: Event-related potential
LFP: Local field potential
PP: Pinprick
S1: Somatosensory cortex
SNR: Signal-to-noise ratio
SSM: State space model
